# A Multiscale Recognition Method for the Optimization of Traffic Signs Using GMM and Category Quality Focal Loss

**DOI:** 10.3390/s20174850

**Published:** 2020-08-27

**Authors:** Mingyu Gao, Chao Chen, Jie Shi, Chun Sing Lai, Yuxiang Yang, Zhekang Dong

**Affiliations:** 1 School of Electronic Information, Hangzhou Dianzi University, Hangzhou 310018, China; mackgao@hdu.edu.cn (M.G.); 15075115@hdu.edu.cn (C.C.); shij@hdu.edu.cn (J.S.); yyx@hdu.edu.cn (Y.Y.); 2 Zhejiang Provincial Key Lab of Equipment Electronics, Hangzhou 310018, China; 3 Department of Electronic and Computer Engineering, Brunel University London, London UB8 3PH, UK; chunsing.lai@brunel.ac.uk; 4 The College of Electrical Engineering, Zhejiang University, Hangzhou 310027, China

**Keywords:** image recognition, traffic sign, Gaussian Mixture Model, multiscale recognition, category imbalance

## Abstract

Effective traffic sign recognition algorithms can assist drivers or automatic driving systems in detecting and recognizing traffic signs in real-time. This paper proposes a multiscale recognition method for traffic signs based on the Gaussian Mixture Model (GMM) and Category Quality Focal Loss (CQFL) to enhance recognition speed and recognition accuracy. Specifically, GMM is utilized to cluster the prior anchors, which are in favor of reducing the clustering error. Meanwhile, considering the most common issue in supervised learning (i.e., the imbalance of data set categories), the category proportion factor is introduced into Quality Focal Loss, which is referred to as CQFL. Furthermore, a five-scale recognition network with a prior anchor allocation strategy is designed for small target objects i.e., traffic sign recognition. Combining five existing tricks, the best speed and accuracy tradeoff on our data set (40.1% mAP and 15 FPS on a single 1080Ti GPU), can be achieved. The experimental results demonstrate that the proposed method is superior to the existing mainstream algorithms, in terms of recognition accuracy and recognition speed.

## 1. Introduction

Traffic accidents occur frequently as a result of drivers’ low attention to the road condition. Conditions including drink driving and fatigue driving jeopardize an individual’s safety and life [1]. Therefore, to reduce the occurrence of accidents and improve the driver’s driving experience, the modes of assisted driving and unmanned driving are paid increasing attention [2]. As an important part of assisted driving and unmanned driving technology, traffic sign recognition technology can recognize traffic signs in real-time while the vehicle is moving, and promptly give instructions or warnings to the driver, or directly control the vehicle to operate [3]. Therefore, the occurrence of traffic accidents can be effectively avoided, and the safety of lives and properties can be guaranteed.

Traffic sign recognition technology cannot recognize traffic signs in real-time without the help of sensor technologies. Sensor technologies can quickly provide clear and undistorted traffic sign images for traffic sign recognition technology when the vehicle is driving fast [4]. In addition, traffic sign recognition technology helps navigation systems to realize route decisions. Put simply, not only is traffic sign recognition a present topic in navigation scenarios, but so are recognition performances in terms of routes themselves [5,6].

However, the traffic sign recognition technology is facing the following challenges:

- The road environment is complicated, which leads to the complicated background of the traffic signs. Even, the traffic signs are partially obscured by other objects.
- The complexity of lighting conditions (including the influence of weather conditions) may cause color distortion of the traffic sign images [7].
- Different shooting angles may cause different degrees of geometric distortion during the collection of traffic sign images [8].
- The color and shape of the traffic signs will change when they are polluted and damaged in nature.

In order to solve the above challenges, corresponding researchers have carried out extensive studies. However, the traditional traffic sign recognition method suffers from the disadvantages of poor recognition performance and network robustness. Although the two-stage traffic sign recognition method (such as modified Faster R-CNN [9]) is proposed, which can achieve high recognition accuracy, its recognition speed is very slow and cannot meet the real-time requirements. The one-stage traffic sign recognition network (such as modified YOLOv3 [10]) can achieve high recognition speed, but its recognition effect is not accurate enough.

The aim of this research is to design an object detector with fast recognition speed, high recognition accuracy and good robustness for traffic recognition system. To this end, this paper proposes a new method of traffic sign recognition, whose contributions can be summarized as follows:

- To the best of the authors’ knowledge, this is the first time that the Gaussian Mixture Model (GMM) [11] is used in prior anchor clustering, which significantly reduces the clustering error and thus improves the recognition accuracy of the neural network and reduces the computational time.
- This paper innovatively adds a category proportion factor in Quality Focal Loss [12] and solves the problem of the poor recognition effect caused by the lack of training samples, which leads to performance improvement.
- The five-scale recognition network and the corresponding prior anchor allocation strategy are proposed, which significantly improves the ability of small target recognition without many extra computing costs.

The rest of this paper is organized as follows. Section 2 introduces the related work. The algorithm description is provided in Section 3. In Section 4, we add some effective tricks to the YOLOv3 [13], setting up a baseline that is much better than origin. Based on this, a prior anchor clustering based on GMM, the Category Quality Focal Loss (CQFL) and a multiscale recognition network with a prior anchor allocation strategy are presented in Section 5. For verification purposes, a series of simulation comparisons with the relevant analysis are conducted and presented in Section 6. Meanwhile, some recognition results on the test set are presented in the same section. Finally, Section 7 concludes the paper.

## 2. Related Work

### 2.1. Traditional Traffic Sign Recognition Algorithm

The traditional recognition algorithms aim to locate out the region of interest and recognize the classification. First, they segment the image by RGB color segmentation [14], HIS color segmentation [15], HSV multi-threshold segmentation [16], etc., and label the connected domain of the binary image. Then, the region of interest is obtained by threshold segmentation [17]. The target region is recognized utilizing template matching [18], SVM [19] and Adaboost [20]. The calculation process of the traditional traffic sign recognition algorithm is simple and easy to implement, which can meet certain recognition requirements. However, the recognition speed is too slow to meet the requirements of real-time recognition and the recognition ability of the recognizer is not good. If affected by the change of light, the accuracy of segmentation may be reduced. Therefore, the speed and accuracy of recognition and robustness of the algorithm need to be improved.

### 2.2. The State-of-the-Art Traffic Sign Recognition Algorithm

Wu et al. [9] propose a two-stage traffic sign recognition method based on modified Faster R-CNN, which absorbs the characteristics of SPP-Net [21] and increases the depth of the network based on R-CNN [22]. Furthermore, it is proposed to use the region recommendation network to extract the recognition area and share the features of the convolution layer with the whole recognition network, which further improves the recognition accuracy. Although it can achieve a certain recognition accuracy, this two-stage recognition algorithm has a slow recognition speed, which cannot meet the real-time requirements.

Based on this situation, the one-stage recognition algorithm is widely studied. Jin et al. [23] propose a traffic sign recognition method based on modified SSD which uses convolutional pyramid feature maps for traffic sign recognition. This algorithm can reuse the multiscale feature maps from different layers calculated in the forward pass. It relies on this characteristic to predict objects with various sizes. Since this algorithm no longer needs to extract the region of interest, its recognition speed is faster. However, this bottom-up pathway suffers from low accuracies on small instances as the shallow-layer feature maps contain insufficient semantic information. To solve the shortcomings of the above algorithm, Feature Pyramid Network (FPN) [24] merges two adjacent feature maps in the backbone model by up sampling the upper layer feature map and then fusing it with the lower layer feature map [25]. These low-resolution feature maps with rich semantic information are combined with high-resolution feature maps with less semantic information to build a feature pyramid that shares rich semantic information at all levels. Based on the FPN, Zhang et al., propose modified RefineDet [26] for traffic sign recognition. This method improves the recognition accuracy to a certain extent, while it loses some speed advantages. Similarly, Branislav et al. [10] propose a traffic sign recognition method based on modified YOLOv3 which takes into account both recognition speed and recognition accuracy. However, this algorithm adopting K-means [13] clustering cannot get accurate prior anchors, resulting in its slow recognition speed and reduced recognition accuracy. Moreover, its three-scale recognition method still cannot meet the recognition requirements of such small targets as traffic signs. Therefore, if this algorithm is used for traffic sign recognition, it may suffer from misdetection and omission issues.

The multiscale recognition method with GMM and CQFL for the optimization of traffic signs solves the common problem of existing traffic sign recognition algorithms. We achieve the best speed and accuracy tradeoff on our data set: 40.1% mAP and 15 FPS on a single 1080Ti GPU.

## 3. Proposed Traffic Sign Recognition Algorithm

**Figure 1:**
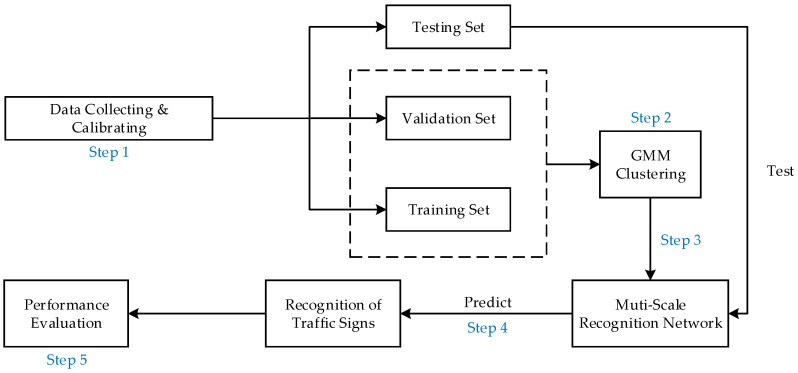
Traffic sign recognition algorithm.

The proposed traffic sign recognition algorithm can be implemented in five steps:

Step 1 Data collecting and calibrating: We collect 10,000 images of traffic lights and traffic signs and divide them into the training set, validation set and testing set. The testing set does not participate in the model training. The calibration task of images is realized by the visual image calibration tool (labelImg).
Step 2 GMM clustering: We cluster all the samples in the training set and the validation set through the GMM to obtain the size of the prior anchors. Details of GMM clustering can be found in Section 5.1.
Step 3 Training the multi-scale recognition network: We take the size of the prior anchors obtained in Step 2 as the parameter of the multi-scale recognition network training, and we add the prior anchor allocation strategy and Category Quality Focal Loss proposed in this paper, as well as some existing effective tricks, to our five-scale recognition network. We train all the training samples and validation samples. The number of training iterations is set to 100. The network parameters are iteratively updated by training. The final training model is obtained once the number of iterations reaches the preset value or the change value of loss function is less than the threshold value.
Step 4 Model testing: Test the final training model obtained in Step 3 on the test set. This model can mark the category and confidence of traffic signs in the correct position of the test image.
Step 5 Performance testing: Objective evaluation of the performance of different traffic sign recognition algorithms (mAP, AP_50_, AP_75_, AP_S_, AP_M_, AP_L_, recall rate, FPS and size of the networks) is calculated and compared. mAP refers to the mean Average Precision when the IoU is constrained to 0.55, 0.6, 0.65, 0.7, 0.75, 0.8, 0.85, 0.9 and 0.95. It is an important metric for judging the accuracy of recognition. The larger its value is, the higher the recognition accuracy is. AP_50_ and AP_75_ is the Average Precision when the threshold of IoU is greater than 0.5 and 0.75. AP_S_, AP_M_ and AP_L_ are the Average Precision of the recognition network in identifying small, medium and large targets. Recall rate is the ratio of the number of positive samples (TP) correctly judged by the recognition network to the number of all positive samples (TP + FN) in the data set. FPS (Frames Per Second) is the number of images that can be recognized by the recognition network per second and is the metric for judging the recognition speed of the network. The larger its value, the faster the speed of recognition [13,23]. Figure 1 presents the holistic process of the traffic sign recognition algorithm.

## 4. Strong Baseline

To compare the existing frameworks, we employ YOLOv3 because of its simplicity and efficiency. YOLOv3 is mainly composed of two parts: a feature extraction network (DarkNet-53) and a feature pyramid network of three levels. In recent years, many scholars have proposed some effective tricks that can significantly improve the performance of YOLOv3 without modifying the network structure and bringing extra inference costs. To better demonstrate the effectiveness of the network structure and tricks proposed in this paper, we built a baseline, much stronger than the origin, based on the existing tricks.

Specifically, the feature extraction network of YOLOv3 is modified: The original activation function (Leaky ReLU) is replaced by the Mish activation function [27], which makes the gradient propagation more efficient and does not take up additional computing resources. The mathematical expression of the Mish activation function can be written by [27]:

(1) $$Mish(x)=x\tanh(\ln(1+e^{x}))$$

Furthermore, the Convolution, Batch Normalization and Mish activation functions form a new convolution structure which serves as the basic unit of the feature extraction network in the Baseline.

In addition, the network training process is improved:

1. To avoid over-fitting, the labeling results are processed by Label Smoothing [28]. The mathematical expression of the Label Smoothing can be written by [28]:
  (2) $$y\_new=y(1-\delta )+\frac{\delta }{NoC},$$
  where, *y_new* denotes the new labeling value after Label Smoothing, *y* denotes the original labeling value, *δ* is a preset coefficient (in general, *δ* = 0.01 [28]) and *NoC* is the number of categories.
  Taking the dichotomies as an example (*NoC* = 2), if the original labeling value *y* = (0, 1), the new labeling value *y_new* is equal to (0.005, 0.995). In other words, Label Smoothing can penalize the labeling results and the baseline uses these penalized labeling results for model training, so that the model cannot be accurately classified during the training process which can avoid overfitting.
2. The Mosaic data enhancement strategy [29] captures four training images which have ground truth bounding boxes at a time and clip the four training images. Then, the trimmed training images can be stitched together according to the preset position. In this process, the four training images and the ground truth bounding box in the training images are combined into one training image. Baseline transforms all the training images according to the Mosaic data enhancement strategy, and it trains the transformed training images. Due to the Mosaic data enhancement strategy, the background of the training samples is enriched. This method is equivalent to increasing the batch size which can improve the recognition accuracy to a certain extent.
3. To solve the divergence problem that may be caused by IoU and GIoU in the training process, we introduce CIoU Loss [30] to measure the gap between the position information of the predicted bounding box and the actual position information. Therefore, CIoU Loss is used as part of the loss function to participate in the training process of baseline. The mathematical expression of the CIoU Loss can be written by [30]:
  (3) $$CL=1-IoU+\frac{\rho^{2}(b,b^{gt})}{c^{2}}+v\alpha$$
  Among them:
  1. *IoU* is the Intersection over Union between the predicted bounding box and the ground truth bounding box
  2. *ρ*^2^ (*b*, *b^gt^*) is the Euclidean Distance between the center of the predicted bounding box and the ground truth bounding box
  3. *c* is the diagonal distance of the smallest closure region that can contain both the predicted bounding box and the ground truth bounding box
  4. *v* denotes the parameter used to measure the consistency of aspect ratio, which is defined as $v\;=\;\frac{4}{\pi^{2}}{(arctan\frac{w^{gt}}{h^{gt}}-arctan\frac{w}{h})}^{2}$
  5. *α* is the parameter used for trade-off, which is defined as $\alpha =\frac{v}{1-\;IoU\;+\;v}$
  CIoU Loss takes into account the distance, overlap rate, scale and penalty terms between the predicted bounding box and the ground truth bounding box, which makes the bounding box regression more stable. As a result, CIoU Loss can achieve better convergence speed and accuracy on the bounding box regression problem.
4. Cosine annealing scheduler [31] is a strategy to adjust the learning rate. After setting the initial learning rate, the maximum learning rate and the number of steps to increase or decrease the learning rate, the value of learning rate first increases linearly and then decreases in line with the cosine function. This pattern of change in learning rate occurs periodically. Cosine annealing scheduler is used to adjust the learning rate of the baseline during training, which helps the model to escape the local minimum during training and find the path to the global minimum by instantly increasing the learning rate.

As shown in Table 1, with the above tricks, the baseline achieves 37.0% mAP on our test set at a speed of 11 FPS (on a single NVIDIA 1080Ti), improving the original YOLOv3-512 (33.1% mAP with 12 FPS) by a large margin without a heavy computational cost.

## 5. Proposed Methodology

### 5.1. Prior Anchors Clustering Based on Gaussian Mixture Model

The use of prior anchors is an effective way to improve the performance of object detection [13]. It cannot only significantly reduce the time required for recognition but also improve the recognition accuracy. Therefore, obtaining an accurate and effective prior anchor in advance becomes the key factor affecting the overall performance of the neural network.

Actually, the elliptical data clusters appear every time in clustering [11,13], while the clustering methods in existing mainstream algorithms (mainly K-means clustering algorithm) cannot cluster the elliptical data clusters. As a result, if the same data are used for clustering, this clustering method eventually makes the clustering results very confusing and result in the decrease of the performance of the recognition network. Hence, the prior anchors clustering based on GMM is proposed in this subsection.

The new clustering method can have an arbitrary ellipse shape in the coordinate system, which is more in line with the actual clustering distribution. Therefore, the flexibility of clustering is improved, and the clustering error is obviously reduced, which can facilitate the autoregression process of the predicted bounding box and reduce the computational time. Specifically, the GMM based prior anchor clustering method is described as below:

The length and width of all the calibration boxes in *n* training samples are extracted as two-dimensional data points to form GMM. With the sample *X_i_*, the mathematical expression of the GMM can be written by [11]:

(4) $$G(X_{i})=\sum_{m=1}^{N}\pi_{m}P(X_{i}|\mu_{m},Var_{m})$$

Among them:

1. *N* is the number of single Gaussian Models in the GMM
2. *π_m_* is the proportion of each single Gaussian Model
3. *P*(*X_i_*|*μ_m_*, *Var_m_*) is the probability density function of the sample *X_i_* in the *m*th single Gaussian Model.

From Equation (4), it can be known that GMM can be formed by superposing some single Gaussian Models with a certain weight ratio. Therefore, GMM can be infinitely approximated by *N* single Gaussian Models. It can be considered that all the calibration box sizes in the training samples can be clustered into *N* different prior anchors with different sizes. To obtain the best clustering effect, the parameters of the GMM need to be estimated by maximizing the likelihood function [11]. In this paper, the Expectation–Maximization Algorithm (EM Algorithm) [32] is used to update the parameters of GMM.

**Step 1:**  Initialize the mean (*μ*), variance (*Var*), and proportion (*π*) of each single Gaussian Model.
**Step 2:**  Calculate the contribution coefficient (*W_i,m_*) of the sample *X_i_* and the initial value of the likelihood function (*L_W_*), respectively:
  (5) $$W_{i,m}=\frac{\pi_{m}P(X_{i}|\mu_{m},Var_{m})}{\sum_{j=1}^{N}\pi_{j}P(X_{i}|\mu_{j},Var_{j})}$$
  (6) $$L_{W}=\sum_{i=1}^{n}\left(\sum_{j=1}^{N}W_{i,j}P(X_{i}|\mu_{j},Var_{j})\right)$$
**Step 3:**  After obtaining *W_i,m_*, the proportion, mean, and variance of each single Gaussian Model in sequence can be updated by Equations (7)–(9) [32]:
  (7) $$\pi_{m}=\frac{\sum_{i=1}^{n}W_{i,m}}{\sum_{i=1}^{n}\sum_{j=1}^{N}W_{i,j}}$$
  (8) $$\mu_{m}=\frac{\sum_{i=1}^{n}W_{i,m}X_{i}}{\sum_{i=1}^{n}W_{i,m}}$$
  (9) $$Var_{m}=\frac{\sum_{i=1}^{n}W_{i,m}{\left(X_{i}-\mu_{m}\right)}^{2}}{\sum_{i=1}^{n}W_{i,m}}$$

**Figure 2:**
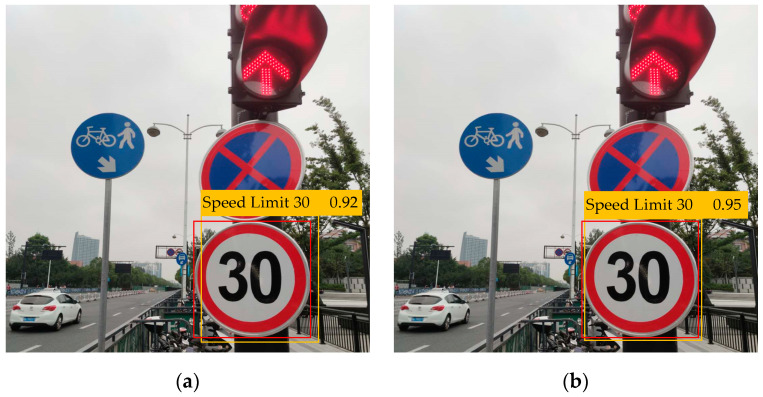
(**a**) is the recognition effect of baseline (K-means), and (**b**) is the recognition effect of the presented method (baseline + GMM). Notably, the ground-truth is labelled by red box, and the predicted one is marked orange. The baseline using the GMM clustering can acquire more accurate location information of the predicted bounding box and improve the recognition accuracy to some extent.

Through the above equations, a process of updating the parameters of the GMM is completed. For each iteration, the updated *W_i,m_* and the likelihood function value can be concurrently achieved by calculating *π_m_*, *μ_m_* and *Var_m_*. This process is iterative. As the parameters are updated, the likelihood function value continues to increase until its value changes less than a preset threshold, at which time the parameters of the GMM reach convergence and the clustering process is completed. The performance improvement of the baseline by GMM is shown in Figure 2.

In our implementation, the predicted results of five scales need to be output (which can be found in Section 5.3). The two smallest scales each need six prior anchors with different sizes, and the remaining three scales each need three prior anchors with different sizes. Therefore, there are 21 different sizes of prior anchors that need to be clustered by the GMM, i.e., *N* = 2 × 6 + 3 × 3 = 21.

### 5.2. Category Quality Focal Loss

In the training process of the network model, the coexistence of difficult-to-train samples and easy-to-train samples is common. However, if the neural network could focus more on the difficult-to-train sample, it can improve the recognition performance of the network. At the same time, for each predicted bounding box obtained in the forward pass, IoU with the ground-truth bounding box can be calculated. If IoU is less than the ignore thresh, the predicted bounding box is regarded as a negative sample, and the penalization is conducted in the network; on the contrary, when the predicted bounding box is judged as a positive sample (i.e., IoU is larger than the ignore thresh), there is no penalty. It can be found that after the network is stable, the ratio of the number of positive and negative samples in a batch is close to 1:1000. Notably, a mass of negative samples may ruin training and degrade model performance.

The emergence of Focal Loss [33] solves the above problems. Focal Loss is improved on the basis of cross-entropy, it is defined as:

(10) $$FL(p_{t})=-\alpha_{t}{\left(1-p_{t}\right)}^{\gamma }\log(p_{t})$$

Among them:

1. $p_{t}=\{\begin{array}{cc} p, & y=1\\ 1-p, & otherwise \end{array}$, *p*∈[0, 1] represents the category prediction probability, and *y* is the label value
2. *CE*(*p_t_*) = −log(*p_t_*) is the cross-entropy function
3. (1 − *p_t_*)*^γ^* is a factor used to control the influencing ability of difficult-to-train samples and easy-to-train samples, *γ* is used to adjust the steepness of the curve
4. *α*∈[0, 1] is a parameter to adjust the influencing ability of positive samples and negative samples.

We call the samples whose category prediction probability *p* is very close to the label value *y* (*y* = 1) as easy-to-train samples. For those easy-to-train samples, the value of (1- *p_t_*)*^γ^* is so small that its influence on Loss is very small. On the contrary, the (1- *p_t_*)*^γ^* of difficult-to-train samples has a larger value, it has a greater influence on Loss. Therefore, we can focus more on difficult-to-train samples.

It should be noted that the value *γ* = 2 and *α_t_* = 0.25 [33] can achieve the best performance in the case of two classifications. In the case of multi-classification, the value is *γ* = 2 and *α_t_* = 1 [33], and Focal Loss is redefined as:

(11) $$FL(p_{t})=-{\left(1-p_{t}\right)}^{\gamma }\log(p_{t})$$

However, Focal Loss is not friendly to us, because it only supports *y* = 1 or *y* = 0. Furthermore, since we use Label Smoothing, *y* is infinitely close to 0 or 1. Therefore, Focal Loss no longer applies. To be used for labels in the form of continuous values, Quality Focal Loss made a small improvement on the basis of Focal Loss to solve this problem. On multi-classification problems, QFL is defined as:

(12) $$QFL(p)=-{\left|y-p\right|}^{\gamma }((1-y)\log(1-p)+y\log(p)),$$

where *p*∈[0, 1] is the category prediction probability, *y* is the label value, and *γ* = 2 [12].

In the process of collecting the data set, we find that the imbalance of the sample size of each category is inevitable. Of course, this phenomenon is also common in supervised learning. As shown in Table 2, it is clear that the recognition results of the neural network for the categories with a small number of samples are poor, which seriously affects the overall performance of the network.

For this reason, we put forward Category Quality Focal Loss (CQFL) on the basis of QFL, which can help the network solve the above-mentioned problems. Furthermore, it is no longer necessary to conduct two separate pieces of training like transfer learning.

For multiple classification problems, the expression of CQFL is:

(13) $$CQFL(p)=-{\left|y-p\right|}^{\gamma }((1-y)\log(1-p)+y\log(p))\beta_{c},$$

where category weight factor *β_c_* determines the importance of category *c* in the model training process. Its mathematical expression can be written by:

(14) $$\beta_{c}=-\ln(\frac{N_{c}}{N}),$$

where *N_c_* is the number of ground-truth labels of category *c* in all training samples, and *N* is the number of ground-truth labels in all training samples.

It can be found that the categories with small sample size usually has a large *β_c_*. Therefore, it can have a greater influencing ability on Loss, allowing the neural network to focus more on these categories. We use CQFL to calculate the difference between the predicted category result and the value of the ground-truth label. Moreover, we add the gap value of the location information obtained by CIoU to get the total Loss during network training. 

To verify the effectiveness of CQFL, relevant experiments are carried out, as shown in Table 3.

Table 3 shows that FL, QFL and CQFL can all improve the performance of the baseline, but CQFL is more capable of improving the performance of the baseline than FL and QFL. Specifically, CQFL can increases the mAP of the baseline by 1.4% without bringing extra inference cost, which is helpful for supervised learning.

### 5.3. Five-Scale Recognition Network with a Prior Anchor Allocation Strategy

To improve the small object recognition performance of the network, we improve the network structure of the baseline and proposes a five-scale recognition network based on FPN.

**Figure 3:**
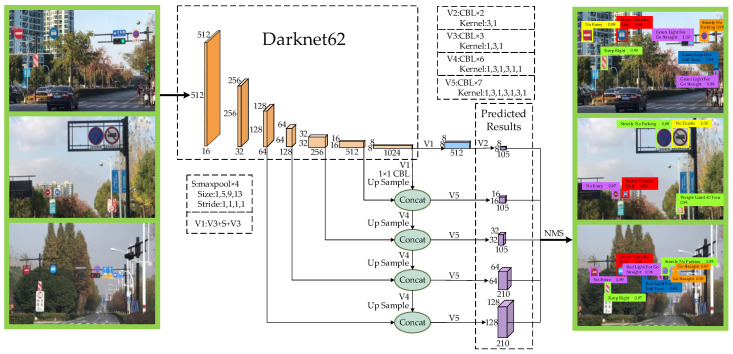
Five-scale recognition network based on FPN. The 128 × 128 × 210 and 64 × 64 × 210 feature maps use small-sized anchors. They have a small receptive field and can have a good recognition effect on small target objects. At the same time, we further improve the performance of the recognition network by up sample and feature fusion.

The recognition network consists of the Backbone (Darknet62) for feature extraction and a five-scale prediction network (as shown in Figure 3). In Darknet62, to prevent over-fitting and increase the nonlinear expression ability of the network, we add Batch Normalization [34] and Mish activation function to the convolutional layer. The CBM (Convolution + Batch Normalization + Mish activation function) convolution is the basic unit of Darknet62. To increase the depth of Darknet62 without the occurrence of gradient explosion, we use a 1 × 1 CBM convolution and a 3 × 3 CBM convolution to perform the residual connect [35] to form a res structure. Furthermore, the res*N* structure is designed with the res structure: a 3 × 3 CBM convolution and *N* res unit structures connected in series.

Darknet62 first resizes the input image to 512 × 512 × 3, and then uses a 3 × 3 CBM convolution to filter the input image. Different from Darknet53, we use res1, res2, res8, res8, res4 and res4 to downsample the feature map in sequence, and at this time it increases the filter of the feature map. Five feature maps of different scales can be obtained sequentially through Darknet62: 128 × 128 × 64, 64 × 64 × 128, 32 × 32 × 256, 16 × 16 × 512, 8 × 8 × 1024. They will be used for the next stage of five-scale prediction.

In the five-scale prediction network, we no longer use the Mish activation function. The CBL (Convolution + Batch Normalization + Leaky ReLU activation function) convolution composed of the convolutional layer, Batch Normalization and Leaky ReLU activation function is the basic unit of the five-scale prediction network. Similarly, we use the CBL convolution to form the res structure and the res*N* structure.

To increase the receptive field in the prediction network, we add the SPP block after the Darknet62. In addition, the SPP block will not bring adverse impacts on the network recognition speed, but it can significantly separate out the most significant context features and improve the network recognition accuracy. Specifically, the SPP block is composed of four max-poolings with different scales (1, 5, 9 and 13) in parallel and then concatenate the outputs. To better match the feature map size and the number of channels, we add three CBL convolutions before and after the SPP block. In a word, we replace the five CBL convolutions of the baseline with three CBL convolutions, the SPP block, and three CBL convolutions in turn.

We adopt the idea of FPN to obtain richer semantic information. Low-resolution but strong semantic feature maps are up sampled and fused with high-resolution but weak semantic feature maps [36] to construct a feature pyramid sharing rich semantics at all levels. Taking the 8 × 8 × 1024 feature map obtained by Darknet62 as an example, the 8 × 8 × 105 predicted result can be obtained through V1 (CBL × 3 + maxpool × 4 + CBL × 3) and V2 (CBL×2). In addition, the 8 × 8 × 1024 feature map also needs to through V1, a 1 × 1 CBL convolution and an up-sample operation to obtain a new feature map which can perform feature fusion with the 16 × 16 × 512 feature map. After feature fusion with the 16 × 16 × 512 feature map, the 16 × 16 × 105 predicted result can be obtained through V5 (CBL × 7). Perform the transformation (as shown in Figure 3) on the remaining three feature maps obtained by Darknet62. Finally, the prediction network can obtain five-scale predicted results: 128 × 128 × 210, 64 × 64 × 210, 32 × 32 × 105, 16 × 16 × 105, and 8 × 8 × 105.

According to [13], the baseline has many anchors to match with the large target object when identifying it. However, for small target objects, even the feature map with the largest resolution in baseline (52 × 52) can only assign three anchors to the small target objects, and their IOU with the ground-truth label is very small. As we all known, the more anchors are matched, the greater the probability of the target being recognized.

**Figure 4:**
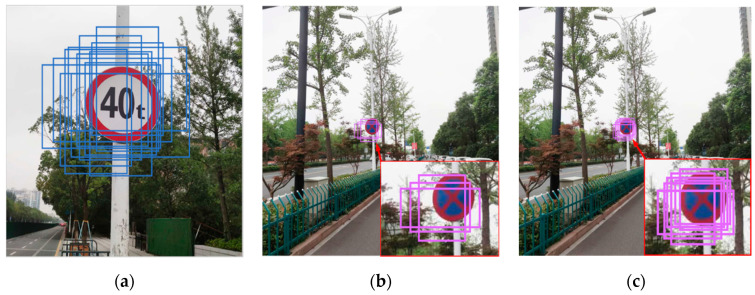
(**a**) shows the anchors matched by the 13 × 13 feature map of the baseline when identifying large target objects. (**b**) shows the anchors matched by the 52 × 52 feature map of the baseline when identifying small target objects. We find that even the feature map with the largest resolution in the baseline can match very few anchors when identifying small target objects. Therefore, the baseline still fails to achieve a good small target recognition effect. (**c**) shows that we assign six anchors of different sizes to the 64 × 64 feature map in the five-scale recognition network, which artificially increases the probability of small target objects being covered by anchors.

To further improve the recognition performance of the recognition network for small objects, we additionally design a prior anchor allocation strategy. As shown in Figure 4, to avoid extra inference costs, we only allocate six anchors with different sizes for the 128 × 128 feature map and 64 × 64 feature map (for each grid), while for the other three scale feature maps, we only assign three anchors. By manually increasing the number of anchors in the feature map, the probability of small target objects being covered by anchors is improved, and the recognition network can obtain a better small target recognition effect.

Combined with the corresponding prior anchor allocation strategy, the five-scale recognition network solves the problem that traffic signs too small to be recognized effectively in the recognition process.

The final predicted results divide the input image into 128 × 128, 64 × 64, 32 × 32, 16 × 16 and 8 × 8 grids, and each grid is assigned a corresponding number of prior anchors. In the process of the forward pass, the recognition network performs a regression process to convert a prior anchor into a corresponding predicted bounding box. Then, the network gets all the predicted bounding boxes together, and removes the redundant predicted bounding boxes through the Non-Maximum-Suppression (NMS) algorithm [37]. Finally, the category of traffic signs can be recognized in the corresponding position of the image or video.

## 6. Experimental Results and Analysis

### 6.1. Traffic Sign Data Set

We conduct statistics on traffic violations in China. And as shown in Table 4, we select 30 kinds of traffic signs with the highest probability of violations as the data set categories.

We collect 10,000 traffic lights and traffic signs images. In the data set, 2000 samples are randomly selected as the test set, not participating in the training of the neural network, but only used to test the performance of the network model. The remain 8000 samples are divided into 6000 training samples and 2000 validation samples; they all need to participate in the training of network models. Our testing set is available at the site [38].

### 6.2. Experimental Environment and Parameter Settings

The experiment environment description: The CPU is Intel^®^ Core™ i7-6700K @4.00GHz, the GPU is GTX1080Ti, the video memory is 11GB, the Windows 10 operating system and the deep learning framework is Tensorflow 1.6.0. We use Python 3.6, OpenCV 3.4.1, and Keras 2.1.5 to achieve traffic sign recognition and corresponding algorithm comparison.

**Figure 5:**
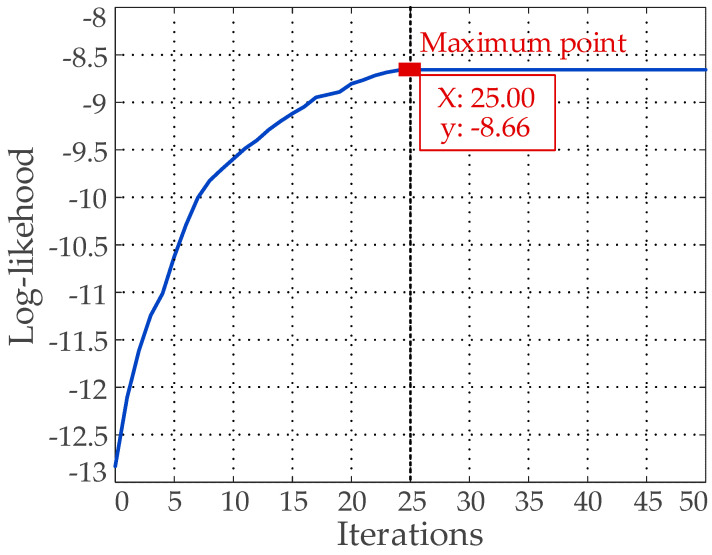
Gaussian Mixture Model (GMM) iteration curve (*N* = 21).

The GMM algorithm is utilized for prior anchor clustering. The parameters of the GMM are adjusted by iterative training so that the likelihood function continuously increases. It should be noted that the value of the likelihood function symbolizes how close the clustering result is to the actual clustering. Correspondingly, the GMM iteration curve (*N* = 21) is shown in Figure 5.

Figure 5 shows that the likelihood function reaches a maximum value at the 25th iteration. Therefore, the experimental results of this iteration are selected as the width and height dimensions of the final 21 prior anchors. Furthermore, the results are assigned to five feature maps of different scales:

1. 128 × 128: (9 × 10), (17 × 20), (21 × 30), (26 × 29), (29 × 47), (25 × 25)
2. 64 × 64: (28 × 32), (33 × 34), (36 × 48), (40 × 42), (33 × 43), (46 × 49)
3. 32 × 32: (48 × 104), (53 × 61), (65 × 68)
4. 16 × 16: (87 × 110), (76 × 82), (100 × 153)
5. 8 × 8: (113 × 134), (162 × 192), (368 × 389)

After obtaining the prior anchors, we can do follow-up experiments.

The parameter setting is provided here. The image size of the data set is adjusted to 512 × 512, and the number of channels of the image is 3. In the iterative process, eight training samples are selected for training each time. The initial learning rate is set to 0.001 and the model parameters are iteratively updated by training to reduce the loss function value. Set the beta to 0.9, momentum to 0.9, and max batches to 500,200. Angle, saturation, exposure, and hue are set to 0, 1.5, 1.5 and 0.1, respectively. It should be noted that the specific parameter settings refer to the existing paper [13].

### 6.3. Traffic Sign Recognition Results and Analysis

**Figure 6:**
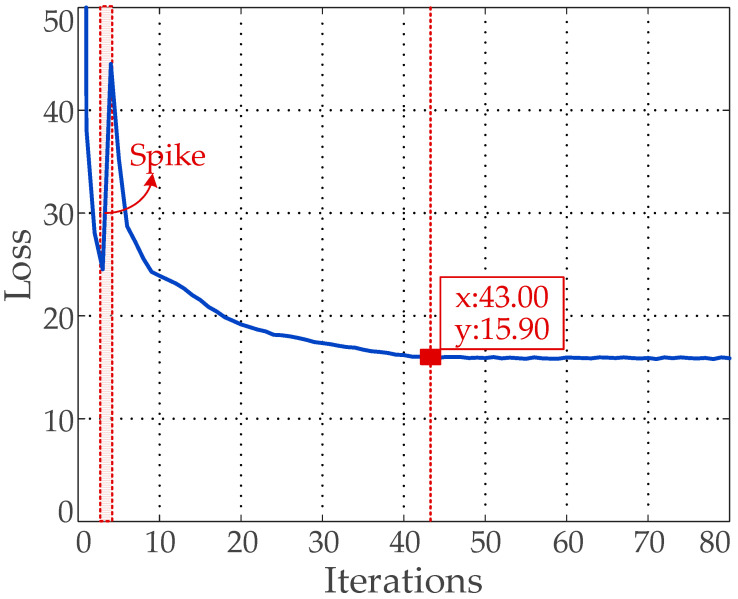
Loss trend curve.

In order to obtain the well-trained model, the number of training is set to 100. During the training phase the network parameters are iteratively updated until the number of iterations reaches the preset value, or the change of loss function is less than the threshold value, and the final training model is achieved. Figure 6 shows that the loss value at the beginning of the model training is very large, but as the training process progresses, the loss value continues to decrease on the whole. Particularly, there is a spike during the 3rd~5th iteration due to the participation of the Cosine annealing scheduler. When the training iterations reach 43 times, the loss tends to be stable, and eventually drops to 15.9 approximately. At this time, the final training model is obtained.

**Figure 7:**
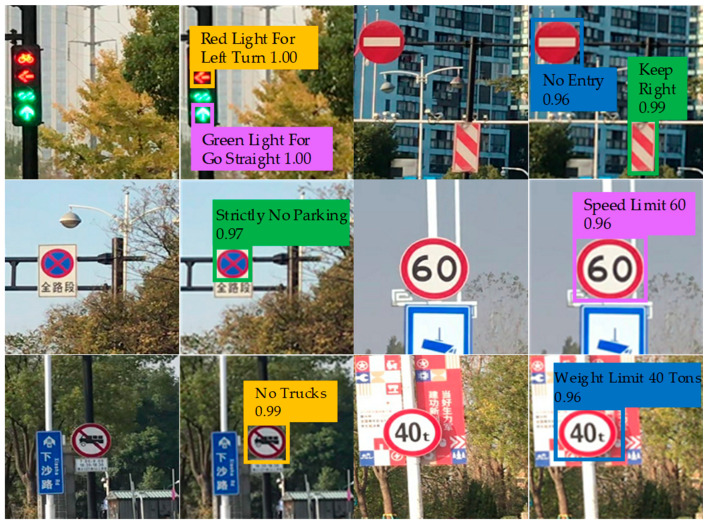
Part of the recognition results.

Figure 7 shows part of the recognition results. It is noted that in the six sets of pictures, the images on the first and third column are the test images, and the remaining images are the recognition results obtained by the proposed algorithm. The recognition results demonstrate that the proposed algorithm is correct and effective.

To illustrate the effectiveness of the proposed tricks from the objective evaluation of performance, we consider applying these four tricks to the baseline (obtained in Section 4) and conduct a series of comparison analysis.

From Table 5, GMM makes the baseline have a better recognition effect on medium targets and large targets and increases the mAP of the baseline by 0.6%. What is more, GMM significantly improves the recognition speed of the baseline by 45.5%. The proposed CQFL can increase the mAP and AP_75_ of the baseline by about more than 1%. This result illustrates the harm of category imbalance to the performance of the recognition network. Besides, the proposed anchor allocation strategy improves the performance of small target recognition of the baseline, which can increase the AP_S_ of the baseline by 1.3%. Furthermore, this strategy can increase the mAP of the baseline by about 0.5%. Last but not least, the proposed five-scale network increases the AP_S_ of the baseline by 1.7%, which means that this network has a good small target recognition capability. Overall, the proposed trick increases the mAP of the baseline by 3.1% and the AP_S_ by 4.0%.

To more effectively illustrate the performance advantages of the proposed traffic sign recognition algorithm, a performance comparison experiment with the state-of-the-art traffic sign recognition algorithms is carried out. It should be noted that to ensure the objectivity of the experimental results, the operating environment of all algorithms is always consistent.

From Table 6, the proposed algorithm achieves a 275% improvement in recognition speed and has greater values of the recall rate, mAP, AP_50_ and AP_75_, compared with the two-stage traffic sign recognition algorithm (modified Faster R-CNN [9]). In addition, the proposed algorithm achieves 3.1% increase in the recall rate, 5.4% increase in the mAP, 3.3% increase in the AP_50_ and 7.4% increase in the AP_75_, compared with one of the strongest competitors in the one-stage traffic sign recognition algorithm (i.e., modified YOLOv3 [10]). Furthermore, the AP_S_ of the proposed algorithm reaches 24.1%, which has 5.5% increase (compare with the modified YOLOv3). Even so, the recognition speed of our algorithm is still 15% faster than the modified YOLOv3. Put simply, the proposed algorithm achieves the best speed and accuracy tradeoff on our data set. However, compared with other traffic sign recognition algorithms, the proposed algorithm takes up too much space, which needs to reduce model parameters by using intelligent optimization methods [39,40,41] in future research.

**Figure 8:**
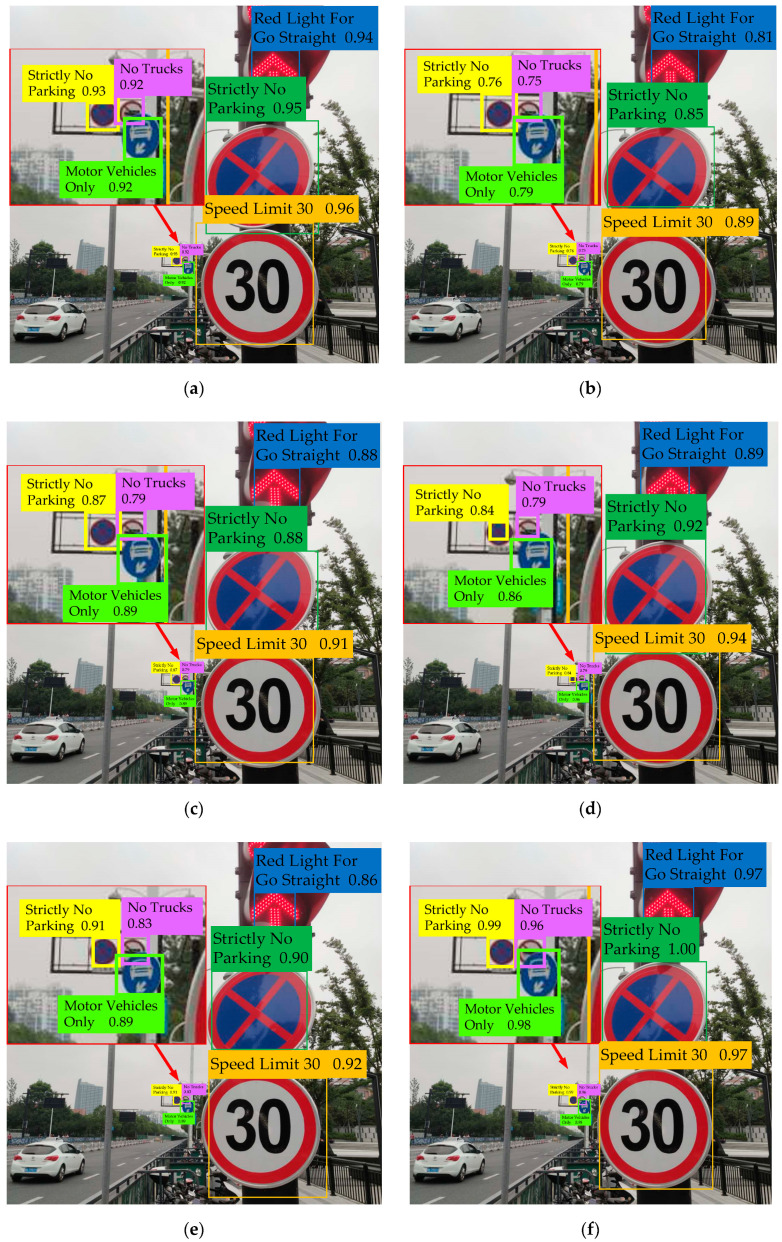
Visual comparison of recognition performance of different traffic sign recognition algorithms for the same image. (**a**–**f**) are the recognition results of the traffic sign recognition algorithms based on modified Faster R-CNN [9], modified SSD [23], modified RefineDet [26], modified RetinaNet101 [39] (its backbone is ResNet-101), modified YOLOv3 [10] and the proposed method.

Moreover, it can be clearly seen from the visual comparison in Figure 8, that the proposed algorithm has certain advantages in recognition accuracy compared with the state-of-the-art algorithms. In particular, our algorithm has a great advantage in the recognition accuracy of small target objects. Specifically, we find that the use of GMM can not only improve the overall recognition accuracy of the network but also shorten the time consumption of the recognition process. By using CQFL to calculate the total loss, we can improve the recognition accuracy of the network for the categories with small sample size. Finally, by using the five-scale recognition network with a prior anchor allocation strategy proposed in this paper, the recognition accuracy of the network for small target objects is significantly improved.

The key reasons that the proposed algorithm can achieve an enhanced performance are:

- Using GMM for prior anchor clustering. The clustering results can have an arbitrary ellipse shape in the coordinate system, which is more in line with the actual clustering distribution. Therefore, the flexibility of clustering is improved, and the clustering error is obviously reduced, which can improve the overall recognition accuracy and speed of the network.
- Using CQFL can make the neural network pay more attention to the categories with small sample size in the training process. Taking its value as a measure of the difference between the predicted category result and the label value, participating in the training of the neural network. It can improve the recognition accuracy of the neural network for the category with small sample size and, therefore, solve the problem of poor recognition effect caused by the small number of samples.
- Based on the proposed recognition network, the resolution of the feature map is increased to 128 × 128, so that the recognition network has a better performance for small objects in the image. Besides, we assign more anchors to 128 × 128 and 64 × 64 feature map, which improves the probability of small target objects being covered by anchors manually. Therefore, the recognition accuracy of the recognition network is further improved, and the problem of small target recognition is solved by our tricks.

## 7. Conclusions

This paper mainly focuses on the investigation of the multiscale recognition method for the optimization of traffic signs. The specific conclusions can be summarized as follows:

1. A scientific traffic sign recognition framework is proposed. The framework is proved by the traffic sign data set containing 30 common traffic sign categories.
2. Based on the existing tricks, we build a baseline with a better performance than the origin. In this paper, the GMM algorithm is used for prior anchor clustering, and a new loss function (CQFL) is proposed based on QFL. Besides, a five-scale recognition network with a prior anchor allocation strategy is proposed. By using the above tricks, the recognition accuracy and recognition speed of the baseline can be significantly improved. Particularly, it has an excellent recognition effect for small target objects.
3. Compared with the state-of-the-art algorithms, the proposed algorithm has certain advantages in recognition accuracy and recognition speed.

Due to the large model parameters, our algorithm and the existing mainstream algorithms suffer from a common issue that the model takes up too much space. In future research, methods such as model pruning and quantization can be used to reduce model parameters and achieve the effect of compressing the model. After implementing model compression, the algorithm in this paper can be better applied to automatic real-time recognition of the traffic sign system.

## Figures and Tables

**Table 1:** The effect of effective tricks in the baseline on recognition performance.

Mish	Label Smoothing	Mosaic	CIoU Loss	Cosine Annealing Scheduler	FPS	mAP	AP_50_	AP_75_
					**12**	33.1%	57.7%	34.1%
✓					11	34.2%	59.0%	35.2%
✓	✓				11	34.4%	59.2%	35.5%
✓	✓	✓			11	34.6%	59.4%	35.9%
✓	✓	✓	✓		11	36.3%	59.7%	38.4%
✓	✓	✓	✓	✓	11	**37.0%**	**60.3%**	**39.2%**

**Table 2:** Recognition accuracy of baseline for some categories.

	Category	Percentage of the Number of Ground-Truth Labels	AP
Small sample size	Yield Ahead	0.05%	1.8%
	One-way Road	0.52%	11.9%
	No U-Turn	1.24%	33.7%
	Bus-only Lane	1.02%	18.8%
	Speed Limit 80	2.06%	35.8%
	Speed Limit 30	1.74%	31.5%
Large sample size	Speed Limit 60	9.95%	82.4%
	No Trucks	8.86%	80.3%
	Keep Right	8.69%	74.7%
	No Entry	9.08%	81.6%
	No Motor Vehicles	8.14%	72.4%
	Stop	7.94%	70.9%

**Table 3:** The performance improvement of the baseline by different loss functions.

Method	FPS	mAP	AP_50_	AP_75_	AP_S_	AP_M_	AP_L_
baseline	11	37.0%	60.3%	39.2%	20.1%	38.2%	48.7%
baseline + FL	11	37.6%	60.6%	39.9%	20.3%	39.0%	49.5%
baseline + QFL	11	37.8%	60.8%	40.3%	20.3%	39.4%	49.8%
baseline + CQFL	11	**38.4%**	**61.2%**	**40.9%**	**20.5%**	**40.9%**	**50.5%**

Note: baseline uses Cross Entropy Loss to calculate the difference between the predicted category result and the value of the ground-truth label.

**Table 4:** Categories of data set collected.

Category				
Traffic Lights	Traffic Signs			
Red Light for Go Straight	Go Straight Slot	No Entry	Bus-only Lane	Speed Limit 120
Green Light for Go Straight	Left Turn Slot	No Trucks	One-way Road	Weight Limit 15 Tons
Red Light for Left Turn	Right Turn Slot	No U-Turn	Motor Vehicles Only	Weight Limit 40 Tons
Green Light for Left Turn	Strictly No Parking	Yield Ahead	Speed Limit 30	Weight Limit 60 Tons
Red Light for Right Turn	No Left or Right Turn	Keep Right	Speed Limit 60	School Crossing Ahead
Green Light for Right Turn	No Motor Vehicles	Stop	Speed Limit 80	Pedestrian Crossing Ahead

**Table 5:** The improvement of the baseline by tricks proposed in this paper.

GMM	CQFL	Anchor Allocation Strategy	Five-Scale Network	FPS	mAP	AP_50_	AP_75_	AP_S_	AP_M_	AP_L_
				11	37.0%	60.3%	39.2%	20.1%	38.2%	48.7%
✓				**16**	37.6%	60.9%	40.6%	20.8%	39.8%	50.1%
✓	✓			**16**	38.9%	61.6%	42.1%	21.1%	42.1%	51.6%
✓	✓	✓		**16**	39.4%	61.9%	42.5%	22.4%	42.3%	51.7%
✓	✓	✓	✓	15	**40.1%**	**63.1%**	**43.8%**	**24.1%**	**43.1%**	**52.2%**

**Table 6:** Objective evaluation of the performance of different traffic sign recognition algorithms.

Method	Size	FPS	Recall Rate	mAP	AP_50_	AP_75_	AP_S_	AP_M_	AP_L_
Method A [9]	109.3M	4	68.9%	39.1%	59.8%	42.7%	18.4%	**43.2%**	50.0%
Method B [23]	100.4M	9	60.3%	29.4%	49.4%	30.6%	10.5%	34.1%	43.6%
Method C [26]	**78.1M**	8	66.3%	33.2%	53.9%	36.3%	15.4%	38.3%	44.9%
Method D [42]	145.6M	6	64.1%	33.6%	52.6%	35.1%	14.4%	37.1%	47.8%
Method E [42]	212.9M	6	67.2%	35.2%	53.9%	37.8%	14.9%	38.7%	49.7%
Method F [10]	237.4M	13	66.4%	34.7%	59.8%	36.4%	18.6%	39.7%	43.8%
Ours	245.3M	**15**	**69.5%**	**40.1%**	**63.1%**	**43.8%**	**24.1%**	43.1%	**52.2%**

Note: Among them, FPS (Frames Per Second) is the metric for judging the recognition speed of the network. The larger its value, the faster the recognition speed of the network. Methods A-F are traffic sign recognition algorithms based on modified Faster R-CNN, modified SSD, modified RefineDet, modified RetinaNet50 (its backbone is ResNet-50), modified RetinaNet101 (its backbone is ResNet-101), modified YOLOv3, respectively. The best results are marked in red.
